# Supplementary material for: The Impact of Maternal and Piglet Low Protein Diet and Their Interaction on the Porcine Liver Transcriptome around the Time of Weaning
Source: Vet Sci. 2021 Oct 14;8(10):233. doi: 10.3390/vetsci8100233 (PMC8540021; doi:10.3390/vetsci8100233)
Supplement: Supplementary file 1 [file vetsci-08-00233-s001.zip › Supplementary figures.pdf]

“The impact of maternal and piglet low protein diet and their interaction on the porcine liver transcriptome around the time of weaning”

# Supplementary figures

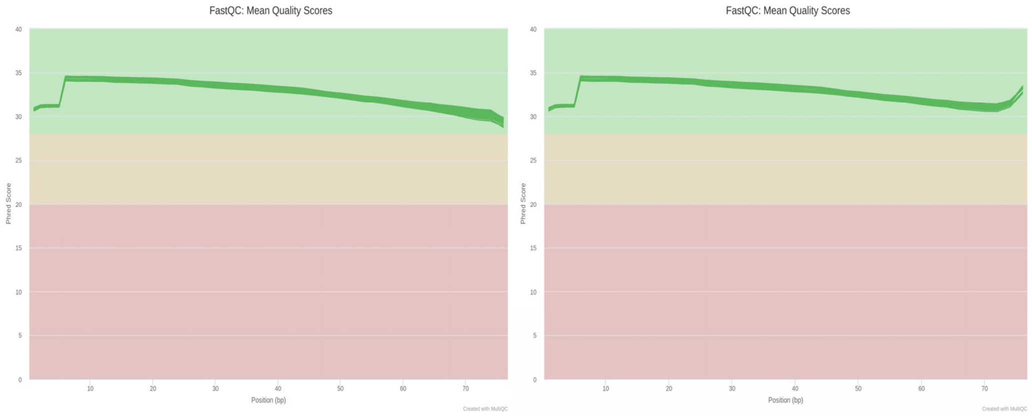


**Figure S1**: Sequence Quality Histograms. Comparison of the Phred score for the raw reads (left) and reads after the adapter and poly(A) sequence trimming (right).

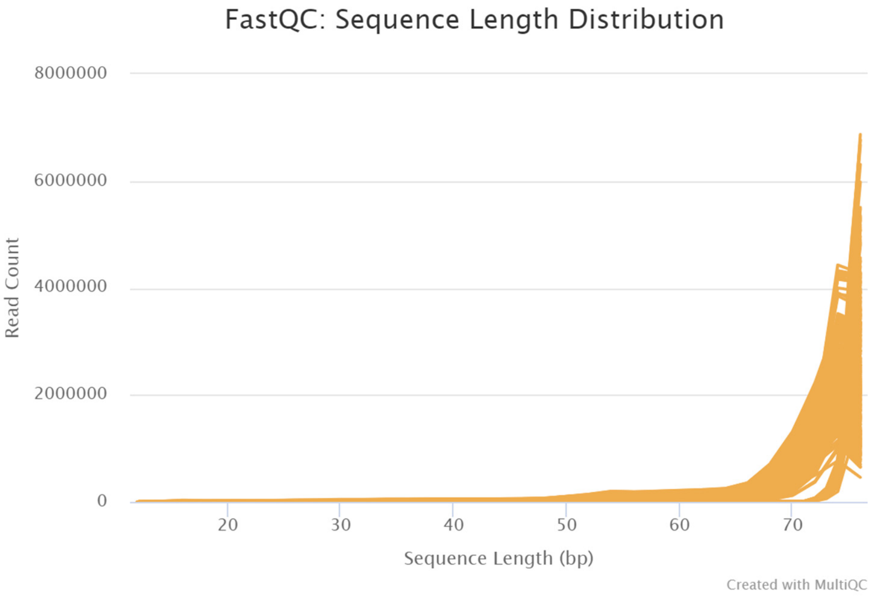


**Figure S2**: Found read length distribution of the reads after trimming adapter and polyA tail sequences.
